# Induced Packaging of Cellular MicroRNAs into HIV-1 Virions Can Inhibit Infectivity

**DOI:** 10.1128/mBio.02125-16

**Published:** 2017-01-17

**Authors:** Hal P. Bogerd, Edward M. Kennedy, Adam W. Whisnant, Bryan R. Cullen

**Affiliations:** Center for Virology and Department of Molecular Genetics and Microbiology, Duke University School of Medicine, Durham, North Carolina, USA; Columbia University

## Abstract

Analysis of the incorporation of cellular microRNAs (miRNAs) into highly purified HIV-1 virions revealed that this largely, but not entirely, mirrored the level of miRNA expression in the producer CD4^+^ T cells. Specifically, of the 58 cellular miRNAs detected at significant levels in the producer cells, only 5 were found in virions at a level 2- to 4-fold higher than that predicted on the basis of random cytoplasmic sampling. Of note, these included two miRNAs, miR-155 and miR-92a, that were reported previously to at least weakly bind HIV-1 transcripts. To test whether miRNA binding to the HIV-1 genome can induce virion incorporation, artificial miRNA target sites were introduced into the viral genome and a 10- to 40-fold increase in the packaging of the cognate miRNAs into virions was then observed, leading to the recruitment of up to 1.6 miRNA copies per virion. Importantly, this high level of incorporation significantly inhibited HIV-1 virion infectivity. These results suggest that target sites for cellular miRNAs can inhibit RNA virus replication at two distinct steps, i.e., during infection and during viral gene expression, thus explaining why a range of different RNA viruses appear to have evolved to avoid cellular miRNA binding to their genome.

## IMPORTANCE

The genomes of RNA viruses have the potential to interact with cellular miRNAs, which could lead to their incorporation into virions, with unknown effects on virion function. Here, it is demonstrated that wild-type HIV-1 virions essentially randomly incorporate low levels of the miRNAs expressed by infected cells. However, the specific incorporation of high levels of individual cellular miRNAs can be induced by insertion of cognate target sites into the viral genome. Of note, this results in a modest but significant inhibition of virion infectivity. These data imply that cellular miRNAs have the potential to inhibit viral replication by interfering with not only viral mRNA function but also virion infectivity.

## INTRODUCTION

The question of how HIV-1 interacts with cellular microRNAs (miRNAs) expressed in infected T cells has been controversial. On the one hand, several groups have reported that a number of different cellular miRNAs bind to specific target sites located on the HIV-1 RNA genome and reduce viral gene expression (1–3), and it has even been suggested that cellular miRNAs can facilitate HIV-1 latency (4). On the other hand, this laboratory has reported that miRNA binding to HIV-1 transcripts, while detectable, is ~100-fold less efficient than miRNA binding to cellular mRNAs expressed contemporaneously in HIV-1-infected T cells (5). This finding is consistent with data demonstrating that the HIV-1 RNA genome is highly structured (6) and that RNA secondary structure inhibits miRNA binding, including to predicted miRNA binding sites present on HIV-1 transcripts (7–9). Moreover, we recently demonstrated that mutational inactivation of human Dicer, which blocks the production of all cellular miRNAs, does not enhance HIV-1 replication or gene expression (10), thus strongly suggesting that HIV-1 has indeed evolved to avoid inhibition by cellular miRNAs.

Another potential way in which HIV-1 might interact with cellular miRNAs is by their specific incorporation into virion particles. Retroviruses are known to package cellular RNAs into virions, and HIV-1 is no exception (11). Among the most abundant cellular RNAs packaged into HIV-1 virions are 7SL RNA and transcripts encoded by various retrotransposons. It has also been suggested that HIV-1 preferentially packages a subset of cellular miRNAs, though how this might affect virion infectivity, if at all, has not been determined (12). Here, we demonstrate that selective miRNA incorporation into HIV-1 virions does occur but is inefficient for wild-type HIV-1. However, miRNA incorporation can be greatly enhanced by insertion into the HIV-1 RNA genome of partially complementary targets specific for individual cellular miRNAs, and this incorporation can significantly inhibit HIV-1 virion infectivity.

## RESULTS

### Wild-type HIV-1 virions incorporate miRNAs in a largely random manner.

**FIG 1:**
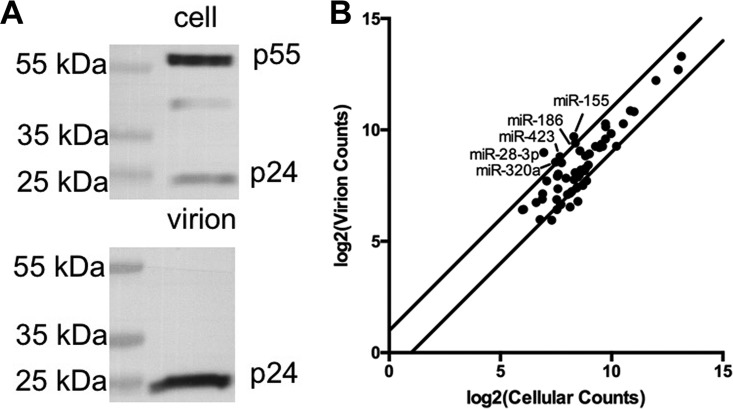
A subset of miRNAs is selectively packaged into HIV-1 virions. (A) Representative Western blot assay of HIV-1 capsid (p24) levels in HIV-1-infected CEM-SS T cells and highly purified virions obtained from the same culture. (B) Most miRNAs are packaged into HIV-1 virions in proportion to their cellular expression level. miRNAs that represent ≥0.1% of the total miRNA level in both purified virions and infected CEM-SS T cells are graphed. The values shown are the averages of data from four independent small RNA-seq experiments. The lines delineate a >2-fold change in relative miRNA levels between virions and cells. Only five cellular miRNAs fall above this line.

To examine whether HIV-1 virions selectively incorporate cellular miRNAs expressed in CD4^+^ T cells, we infected CEM-SS T cells with HIV-1, waited 72 h, and then harvested both the producer CEM-SS cells and the supernatant medium. After filtration, virions were purified from the medium by pelleting through a sucrose cushion, followed by centrifugation through a 6 to 18% OptiPrep gradient. This protocol has been shown to effectively separate HIV-1 virions from cellular exosomes (13). Analysis of the CEM-SS cell and virion fractions by Western blotting for the HIV-1 p24 protein revealed high levels of the uncleaved p55 Gag polyprotein in producer cells and exclusively mature p24 capsid protein in the purified virion sample, as expected (Fig. 1A). Transcriptome sequencing (RNA-seq) was then performed on the small RNA fraction (15 to 30 nucleotides [nt]) recovered from the producer cells and virions and the average of four independent small RNA-seq experiments, two analyzing miRNAs in purified virions and two analyzing miRNAs from the matched producer cells, is shown in Fig. 1B. Shown are the 58 miRNAs that are detected in both CEM-SS cells and HIV-1 virions at a level of ≥0.1% of the total miRNA pool, a cutoff based on data showing that miRNAs expressed below this level are not functionally relevant (14).

As shown, there was generally a good correlation between the relative level of each cellular miRNA detected in producer cells and in virions, with only 5 of the 58 miRNAs analyzed found at a >2-fold higher level in virions than in producer cells (Table 1). Of note, of the 20 miRNAs most highly expressed in CEM-SS cells, none were incorporated >1.5-fold more into virions than predicted on the basis of random sampling of the cytoplasm (see Table S1 in the supplemental material).

**TABLE 1:** Identities of cellular miRNAs that are selectively incorporated into HIV-1 virions^a^

miRNA	Expression in cells (%)			Virion incorporation (%)			Ratio
	Rep^b^ 1	Rep 2	Avg	Rep 1	Rep 2	Avg	
miR-28-3p	0.122	0.373	0.248	0.902	1.126	1.014	4.1
miR-155-5p^c^	0.683	0.579	0.631	1.688	1.647	1.668	2.6
miR-423-3p^c^	0.283	0.534	0.409	0.751	1.035	0.893	2.2
miR-320a-3p	0.502	0.211	0.357	1.027	0.471	0.749	2.1
miR-186-5p	0.388	0.935	0.662	1.486	1.234	1.360	2.1

a Listed are the cellular miRNAs, expressed at ≥0.1% of the total miRNA pool in CEM-SS cells, that are, on average, incorporated >2-fold more effectively into HIV-1 virions than predicted on the basis of their expression in infected producer cells.

b Rep, replicate.

c miRNA previously reported (5) to bind to sites in the HIV-1 genome.

10.1128/mBio.02125-16.1 TABLE S1  Relative expression of the 20 most highly expressed miRNAs in HIV-1 infected CEM-SS cells, and purified HIV-1 virions derived from these cells. The data shown are percentages of the total miRNA assigned reads, for both infected CEM-SS cells and purified HIV-1 virions, determined by small RNA-seq. We obtained between 3.4 × 10^7^ and 2.6 × 10^7^ total small RNA reads in each of the four libraries analyzed, of which between 2.6 × 10^7^ and 2.1 × 10^7^ reads could be aligned with the human genome. Of these, between 9.9 × 10^6^ and 4.6 × 10^6^ reads could be aligned with known human miRNAs. Download TABLE S1, DOCX file, 0.02 MB. Copyright © 2017 Bogerd et al. 2017 Bogerd et al. This content is distributed under the terms of the Creative Commons Attribution 4.0 International license.

While the small number of miRNAs found to be selectively incorporated into virion particles argues that miRNA incorporation is largely random, we were intrigued by the identity of these miRNAs. Specifically, miR-155 and miR-423-3p are two of the four cellular miRNAs that we previously reported are able to specifically, albeit inefficiently, bind to partially complementary target sites on the HIV-1 RNA genome (5). While miR-28 has also been reported to bind the HIV-1 genome (4), we note that that paper was actually referring to miR-28-5p, the miRNA derived from the other arm of pre-miR-28, so that earlier report does not provide an explanation for why miR-28-3p is concentrated into HIV-1 virions. In fact, we observed low but identical levels of miR-28-5p (0.05% of the total miRNA reads) in both the CEM-SS T cells and purified virions analyzed. We note that the proposed target site for miR-28-5p (4) does not have seed homology to this miRNA, and RNA induced silencing complex (RISC) binding to this site was not detected in our previous analysis of miRNA binding to the HIV-1 genome (5).

The observation that two miRNAs previously reported to bind the HIV-1 genome (5) are packaged into virions at higher-than-expected levels suggested that selective miRNA incorporation might be mediated by the binding of miRNA-programed RISCs to complementary viral target sites. To test this idea, we generated indicator HIV-1 strains in which the nanoluciferase (NLuc) gene was inserted at the beginning of the *nef* open reading frame (ORF), followed 3′ by two tandem miRNA target sites or by an equivalent random (RAN) sequence lacking significant complementarity to any human miRNA (see Fig. S1 in the supplemental material). Thus, every mRNA expressed by these viruses, including the NLuc mRNA, would contain the inserted sequence in their 3′ untranslated region. The inserted miRNA targets were designed to be partially complementary to miR-92a or miR-155, with two central mismatches that have been previously shown to block mRNA cleavage by RISC (15, 16). miR-92a is the third most highly expressed miRNA in CEM-SS cells, contributing ~8.2% of the entire miRNA pool (see Table S1), while miR-155 contributes ~0.63% of the miRNA pool (Table 1). While miR-92a is also highly expressed in 293T cells (see Fig. S2A), these cells do not express miR-155 (see Fig. S2B). To express miR-155 in 293T cells, we transfected them with a vector that expresses pri-miR-155, and hence mature miR-155, at a level comparable to that seen in CEM-SS cells (see Fig. S2B).

10.1128/mBio.02125-16.2 FIG S1  Schematic of the replication-competent, NLuc-expressing HIV-1 indicator viruses used in these experiments. The NLuc ORF was inserted at the 5′ end of *nef*, and miRNA target sites were inserted 3′ to NLuc. Download FIG S1, TIF file, 4 MB. Copyright © 2017 Bogerd et al. 2017 Bogerd et al. This content is distributed under the terms of the Creative Commons Attribution 4.0 International license.

10.1128/mBio.02125-16.3 FIG S2  Levels of miR-92a and miR-155 expression in CEM-SS and 293T cells. Total RNA was isolated from uninfected CEM-SS, 293T+CD4/CXCR4, and 293T+CD4/CXCR4/miR-155 cells, and miRNA and endogenous U6 RNA levels were determined with a TaqMan qRT-PCR assay. The values shown are normalized to the U6 control. (A) The level of miR-92a detected in CEM-SS cells was set to 1. Relative levels of endogenous miR-92a in 293T cells with or without miR-155 are shown. (B) Similar to panel A except that miR-155 levels were measured with the level detected in CEM-SS cells set to 1. Relative levels of miR-155 expression in wild-type 293T cells, which do not express detectable miR-155, and 293T cells ectopically expressing miR-155, are shown. The data shown are from three independent experiments with standard deviations indicated. Download FIG S2, TIF file, 5.7 MB. Copyright © 2017 Bogerd et al. 2017 Bogerd et al. This content is distributed under the terms of the Creative Commons Attribution 4.0 International license.

### Target sites on the HIV-1 RNA genome promote virion incorporation of miRNAs.

**FIG 2:**
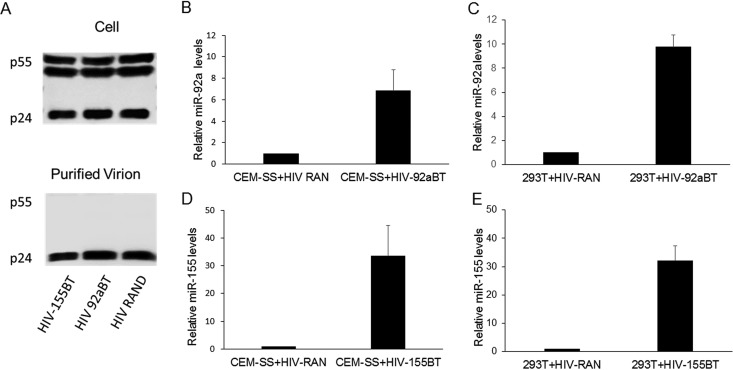
Relative miRNA and HIV-1 RNA levels in purified HIV-1 virions measured by quantitative PCR. Virions were collected at 72 h postinfection. Fold changes in the levels of packaged miR-92a and miR-155, relative to HIV-1 genomic RNA, were determined by the ΔΔ*C*_*T*_ method (30). An HIV-1 clone bearing a randomized miRNA target (RAN) served as the negative control and was set to 1. (A) Western blot assay to assess the purity of HIV-1 virions. (Top) p24 Western blot assay of HIV-1-infected CEM-SS cell lysates. (Bottom) p24 Western blot assay of purified virions. (B) Packaging of miR-92a into HIV-1 virions containing two miR-92a BTs produced by CEM-SS T cells. (C) Packaging of miR-92a into HIV-1 virions containing two miR-92a BTs produced by 293T cells. (D) Packaging of miR-155 into HIV-1 virions containing two miR-155 BTs produced by CEM-SS cells. (E) Packaging of miR-155 into HIV-1 virions containing miR-155 BTs produced by 293T cells expressing ectopic miR-155. The data shown are from three to five independent experiments with standard deviations indicated.

To examine whether the presence of target sites indeed facilitates miRNA virion incorporation, we infected CEM-SS cells, wild-type 293T cells, or miR-155-expressing 293T cells with HIV-1 proviruses containing the two partially mismatched bulged target (BT) sites for miR-92a (HIV-92aBT) or miR-155 (HIV-155BT). We then harvested the producer cells and recovered purified HIV-1 virions from the supernatant medium as described above. We again detected unprocessed p55 Gag in the producer cells but exclusively fully processed p24 in the purified virions (Fig. 2A). Importantly, we observed an 8- to 10-fold enrichment of virion-incorporated miR-92a in HIV-92aBT virions derived from both CEM-SS and 293T cells, compared to control HIV-RAN virions, as measured by TaqMan quantitative reverse transcription (qRT)-PCR (Fig. 2B and C). Even more impressively, HIV-155BT virions produced from CEM-SS cells or 293T cells expressing ectopic miR-155 incorporated 30- to 40-fold higher levels of miR-155 than did the control HIV-RAN virions (Fig. 2D and E). The increased virion incorporation of these miRNAs was highly statistically significant (*P* < 0.005). Interestingly, we saw a modest reduction in miR-155 expression in HIV-1-infected CEM-SS cells that approached statistical significance (*P* = 0.06) (see Fig. S3A), although the miR-92a expression level did not appear to be affected by HIV-1 infection (see Fig. S3B). The mechanistic basis of this reduction, which was not affected by the introduction of miR-155 target sites into the HIV-1 genome, is unclear. Surprisingly, the introduction into the HIV-1 genome of tandem target sites for miR-155 and miR-92a did not affect the level of viral RNA expressed in infected CEM-SS cells (see Fig. S3C), even though the dramatic increase in virion incorporation of these miRNAs argues that these target sites are indeed bound by their cognate miRNAs.

10.1128/mBio.02125-16.4 FIG S3  Relative levels of miRNA and viral RNA expression in CEM-SS cells. RNA levels in HIV-1-infected CEM-SS cells were determined by TaqMan qRT-PCR, and values were then normalized to the U6 RNA endogenous control by the ΔΔ*C*_*T*_ method. (A) The level of miR-155 expression in uninfected CEM-SS cells (control [Ctrl]) was set to 1. The relative levels of miR-155 in CEM-SS cells infected with HIV-155BT, HIV-92aBT, and HIV-RAN are shown. (B) The level of miR-92a expression in uninfected CEM-SS cells (Ctrl) was set to 1. The relative levels of miR-92a in CEM-SS cells infected with HIV-155BT, HIV-92aBT, and HIV-RAN are shown. (C) The levels of HIV-1 RNA in cells infected with HIV-155BT, HIV-92aBT, and HIV-RAN were determined with a TaqMan probe specific for the *pol* gene. The level of HIV-1 RNA in HIV-RAN-infected cells was set to 1, and the relative levels of HIV-155BT and HIV-92aBT are shown. Ctrl represents cells that were incubated with supernatant medium from 293T cells transfected with a replication-incompetent HIV-1 proviral clone containing an intact *pol* gene to control for plasmid DNA carryover. The data shown are from three independent experiments with standard deviations indicated. Download FIG S3, TIF file, 4.1 MB. Copyright © 2017 Bogerd et al. 2017 Bogerd et al. This content is distributed under the terms of the Creative Commons Attribution 4.0 International license.

**FIG 3:**
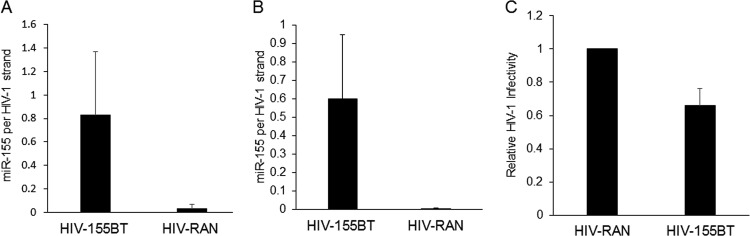
Quantification of HIV-1 genomic RNA and miR-155 levels in virions. Standard curves for both miR-155 and the HIV-1 *pol* gene were generated by TaqMan qRT-PCR. Experimental values for miR-155 and HIV-1 RNA levels in total RNA isolated from purified HIV-1 virions were measured, and the absolute numbers of each molecule were determined. (A) Number of miR-155 copies per HIV-1 genomic RNA in virions produced from CEM-SS T cells infected with HIV-miR-155BT or with the HIV-RAN control. (B) Similar to panel A except that the number of miR-155 copies per HIV-1 genomic RNA were determined in virions produced by 293T cells expressing ectopic miR-155. (C) Inhibition of HIV-1 infectivity by miR-155 packaged into HIV-1 virions. Pseudotyped virions (HIV-RAN and HIV-155BT) were produced in 293T cells expressing ectopic miR-155, and equivalent amounts of virus, as determined by p24 level, were used to infect naive 293T cells. Cells were lysed at 20 h postinfection, and NLuc levels were determined. The data shown are from three independent experiments with standard deviations indicated.

To determine the actual level of incorporation of miR-155 into HIV-1 virion particles, we performed TaqMan qRT-PCR to determine the number of miR-155 molecules per viral RNA genome in purified virion particles. This analysis revealed that the insertion of tandem miR-155 target sites increased the number of miR-155 strands per HIV-1 genome from 0.03 ± 0.03 to 0.83 ± 0.54 in virions, a dramatic and statistically significant (*P* = 0.015) change (Fig. 3A). Similarly, in 293T cells expressing miR-155, the level of virion-incorporated miR-155 increased from essentially undetectable to ~0.6 miR-155 strand per HIV-1 genome (Fig. 3B), that is, to ~1.2 miR-155 molecules per diploid virion. To examine whether this dramatic increase in miRNA incorporation would affect virion infectivity, we infected naive, miR-155-negative 293T cells with identical levels of HIV-155BT or control HIV-RAN virions produced in miR-155-expressing 293T cells. Remarkably, we observed that the HIV-155BT virions were indeed ~30% less infectious than the control HIV-RAN virions and this difference was significant (*P* = 0.029) (Fig. 3C). This result demonstrates that the target site-mediated incorporation of cellular miRNAs can directly inhibit HIV-1 virion infectivity.

### Introduced miRNA target sites induce a modest inhibition of HIV-1 gene expression.

While virion incorporation of miR-155 at high levels reduces virion infectivity (Fig. 3C), we were surprised that the introduction of two tandem BTs for miR-92a or miR-155 did not detectably affect the level of HIV-1 transcripts in infected CEM-SS cells (see Fig. S3C), even though these same targets clearly induced the virion incorporation of their cognate miRNAs (Fig. 2B) and must therefore bind these miRNAs.

To examine inhibition of HIV-1 gene expression by cellular miRNAs in more detail, we constructed two additional reporter viruses containing fully complementary perfect targets (PTs) for miR-155 and miR-92a to test alongside the viruses containing the partially mismatched BTs for the same two miRNAs discussed above. Because targets perfectly complementary to a miRNA are subject to cleavage by RISCs containing Ago2, we would predict greater inhibition of the PT indicator viruses than of the BT viruses.

**FIG 4:**
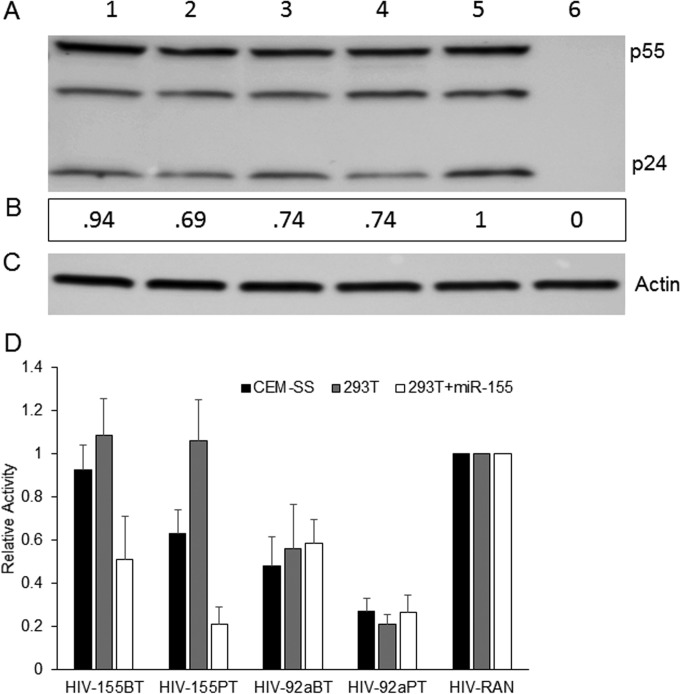
Effect of inserted miRNA BTs or PTs on HIV-1 replication. (A) Representative p24 Western blot assay of whole-cell lysates derived from 293T cells producing HIV-NLuc-155BT (lane 1), HIV-NLuc-155PT (lane 2), HIV-NLuc-92aBT (lane 3), HIV-NLuc-92aPT (lane 4), or HIV-NLuc-RAN (lane 5) or from uninfected cells (lane 6). (B) Relative p24 levels determined by Gene Tools software (Syngene). (C) β-Actin internal loading control. (D) NanoLuc levels in CEM-SS, 293T+CD4/CXCR4, and 293T+CD4/CXCR4/miR-155 cells infected with an HIV-155BT, HIV-155PT, HIV-92aBT, HIV-92aPT, or HIV-RAN preparation. The NLuc activity induced upon pNL-NLuc-RAN infection was set to 1, and the relative NLuc levels in CEM-SS, 293T+CD4/CXCR4, and 293T+CD4/CXCR4/miR-155 cells infected with HIV-NLuc-155BT, HIV-NLuc-155PT, HIV-NLuc-92aBT, or HIV-NLuc-92aPT were determined. The data shown in panel D are from four independent experiments with standard deviations indicated.

To perform this experiment, we generated stocks of HIV-92aBT, HIV-92aPT, HIV‑155PT, HIV-155BT, and the control HIV-RAN indicator by transfection of wild-type 293T cells. Similar levels of all five viruses were generated, as assessed by Western blotting of the producer cells for p24 (Fig. 4). The resultant viral stocks were used to infect CEM-SS cells, wild-type 293T cells, or 293T cells expressing ectopic miR-155. CEM-SS and 293T cells both express high levels of miR-92a, while the level of miR-155 is ~10-fold lower (Table 1; see Table S1 and Fig. S2). As expected, we did not observe any inhibition of NLuc expression from HIV-155BT in wild-type 293T cells, which do not express miR-155 (Fig. 4D), thus further supporting the hypothesis that the inhibitory effect seen in Fig. 3C was at the level of virion infectivity. Surprisingly, HIV-155BT also was not detectably inhibited in CEM-SS cells, though we did see a modest but significant (*P* = 0.015) ~2-fold inhibition in 293T cells expressing ectopic miR-155. Analysis of NLuc expression from the virus containing two miR-155 PT sites, HIV-155PT, revealed no inhibition in miR-155-negative wild-type 293T cells and a marked ~5-fold drop in NLuc expression in 293T cells expressing ectopic miR-155 (*P* = 0.0002). For HIV-155PT, we also saw an ~2-fold reduction in NLuc expression in CEM-SS cells that approached statistical significance (*P* = 0.06). As expected, given the higher level of expression of miR-92a relative to miR-155 in both CEM-SS and 293T cells, HIV-92aBT was inhibited by ~2-fold, while HIV-92aPT was inhibited by ~4-fold, in both cell types. Therefore, while we did indeed see inhibition of HIV-1 gene expression when miRNA targets complementary to endogenous or ectopically expressed miRNAs were introduced into the HIV-1 genome, this inhibition is modest when these targets are only partially complementary.

## DISCUSSION

Previously, we have reported that the interaction of cellular miRNAs with HIV-1 transcripts is very inefficient, though weak binding of a few cellular miRNAs, including miR-155 and miR-423-3p, could be detected (5). Consistent with the idea that HIV-1 has evolved to avoid the binding of miRNA-programed RISCs to viral mRNAs, and hence any resultant translational repression and/or destabilization, we have also demonstrated that blocking miRNA biogenesis entirely, by mutational inactivation of Dicer, does not detectably enhance the replication of HIV-1 or indeed a wide range of other RNA viruses (10), a result confirmed by others (17). Here, we have extended this earlier work by asking if cellular miRNA binding to the genome of HIV-1 has any phenotypic effects. Analysis of the level of cellular miRNA packaging into highly purified wild-type HIV-1 virions revealed a close correlation with the miRNA expression level in the producer T cells and appeared largely random (Fig. 1). We did, however, identify a small number of cellular miRNAs that were slightly overrepresented in virions (Table 1) and, interestingly, these included miR-155 and miR-423-3p, which we had previously reported to weakly interact with HIV-1 transcripts (5). To test whether selective miRNA incorporation into virions could be enhanced by their recruitment to the HIV-1 genome, we introduced artificial target sites for two cellular miRNAs, miR-155 and miR-92a (see Fig. S1), and this indeed resulted in a dramatic increase in their incorporation into virion particles (Fig. 2B to E). Precise quantitation by TaqMan qRT-PCR revealed that the introduced miR-155 target sites induced the packaging of ~1.2 to ~1.6 miR-155 molecules per virion particle (Fig. 3A and B), which in turn reduced HIV-1 virion infectivity significantly (Fig. 3C). Therefore, while inserted miRNA target sites can clearly inhibit HIV-1 gene expression at the level of viral mRNA function (Fig. 4), these same miRNA target sites can also induce the selective incorporation of miRNAs into HIV-1 virion particles and, hence, directly inhibit their infectivity. At what level this inhibition occurs is not known. However, it seems possible that a miRNA-programed RISC bound to the viral genome might have the potential to inhibit RT.

We note that there has been considerable interest in using the insertion of targets for cellular miRNAs into viral genomes with the goal of controlling viral tissue tropism or generating attenuated vaccines (18–20). While it has been generally assumed that the reduced replication of these viruses in cells expressing the cognate miRNA is exclusively due to reduced viral mRNA function, our data suggest that the inserted targets for cellular miRNAs can also affect viral fitness by directly inhibiting virion infectivity. It will therefore be of interest to test whether cellular miRNAs can indeed be selectively incorporated into virions produced by positive-sense RNA viruses other than retroviruses. Of note, while cellular miRNA binding to viral RNAs, if it did occur, would likely be inhibitory to the large majority of viral species, at least two viruses, the human hepacivirus hepatitis C virus (HCV) and the distantly related animal pestivirus bovine viral diarrhea virus (BVDV), have been shown to require specific cellular miRNAs for their effective replication (21, 22). These cellular miRNAs, miR-122 in the case of HCV and miR-17 and let-7 in the case of BVDV, exert this still poorly understood positive effect via a direct interaction with the viral RNA genome. This raises the possibility not only that these miRNAs might be specifically packaged into HCV or BVDV virions but also that such packaged miRNAs might actually facilitate the early steps of the replication cycle of these viruses.

## MATERIALS AND METHODS

### Molecular clones.

The previously published HIV-1 proviral expression plasmid pNL-Luc-HXB (23) was modified as follows. The firefly luciferase ORF was removed by NotI-XhoI digestion and replaced with the NLuc ORF (Promega) to generate pNL-NLuc-HXB. Oligonucleotides encoding two fully complementary miRNA PT sites for miR-155 or miR-92a, or BTs mismatched at two adjacent central nucleotides (see Fig. S4), were annealed and inserted into the unique XhoI site 3′ of NLuc to generate pNL-NLuc-155BT (HIV-155BT), pNL-NLuc-155PT (HIV-155PT), pNL-NLuc-92aBT (HIV-92aBT), and pNL-NLuc-92aPT (HIV-92aPT). A similar DNA fragment of a RAN sequence was inserted to generate pNL-NLuc-RAN. Two miR-155 expression vectors were used in this work. The pcDNA-based pmiR-155 expression plasmid was used in the transient expression experiments shown in Fig. 2 and 4, while a murine stem cell virus-based retroviral vector (pMSCV-miR-155) was used to generate the stable miR-155-expressing 293T cell clone used in Fig. 3B and C. HIV-1 coreceptor expression plasmids pCMV/CD4 and pCMV/CXCR4 have been previously described (24).

10.1128/mBio.02125-16.5 FIG S4  miRNA target sequences inserted into the HIV-1 genome. Lowercase bases represent a linker sequence inserted between the two tandem miRNA target sites. Bold bases indicate mismatches inserted into the BT sites. Download FIG S4, PDF file, 0.03 MB. Copyright © 2017 Bogerd et al. 2017 Bogerd et al. This content is distributed under the terms of the Creative Commons Attribution 4.0 International license.

### Cell culture.

293T cells were maintained in Dulbecco’s modified Eagle’s medium supplemented with 5% fetal bovine serum (FBS) and gentamicin (Gibco). CEM-SS cells (25) (catalog no. 776; NIH AIDS Reagent Program) were maintained in RPMI supplemented with 10% FBS and gentamicin. Cells were confirmed to be mycoplasma negative.

293T cells stably expressing miR-155 were generated by transfection of 10 µg of pMSCV-miR-155 and 2 µg of pVSV-G into 2 × 10^6^ 293T cells by the polyethylenimine (PEI) method. Seventy-two hours posttransfection, the medium was collected and filtered and naive 293T cells were transduced. Forty-eight hours posttransduction, the cells were selected with 2 µg/ml puromycin and then single cell cloned. A clone expressing miR-155 was then identified by TaqMan qRT-PCR and used in the experiments shown in Fig. 3.

### Sequencing of miRNA from HIV-1-infected CEM-SS cells and purified virions.

HIV-1 was produced by transfecting 10 µg of pNL-NLuc-RAN into 2 × 10^6^ 293T cells with PEI. Seventy-two hours posttransfection, virus-containing supernatant medium was harvested, passed through a 0.45-µm filter, and used to infect CEM-SS cells. Twenty-four hours postinfection, the cells were washed twice with phosphate-buffered saline (PBS) and the medium was replenished. At 72 h postinfection, the culture was centrifuged and the cells were harvested. The supernatant medium was passed through a 0.45-µm filter, layered over a 20% sucrose cushion in PBS, and centrifuged at 40,000 rpm for 2 h. The pelleted virions were resuspended in PBS and layered over a 6 to 18% OptiPrep (Axis-Shield) gradient (13) and centrifuged a second time. The virion-containing OptiPrep fractions (14.4 to 18%) were collected and diluted in PBS, and the virions were pelleted. The total RNA was extracted from the purified virions and from the infected CEM-SS producer cell pellet with TRIzol (Thermo Fisher Scientific). Small RNA cDNA libraries were then constructed essentially as described previously (26), with an Illumina TruSeq small-RNA kit, prior to sequencing with an Illumina HiSeq 2000.

### Bioinformatic analysis of small RNA-seq data.

Small RNA reads of ≥15 nt were collapsed into FASTA format with the FASTX toolkit (http://hannonlab.cshl.edu/fastx_toolkit/index.html) by using the following pipeline: fastq_quality_filter -Q33 | fastx_clipper -a TruSeq-Indexnumber -l 15 − c | fastq_to_fasta -Q33 | fastx_collapser. All reads were then subjected to alignment with Bowtie version 0.12.7 with the following options: –a –best –strata -m 25. Sequences were sequentially filtered and assigned by using the following pipeline (displayed as database, additional Bowtie alignment parameters): (i) 3′ and 5′ adapters, -v 0 –noRC; (ii) HIV genome, -v 1; (iii) miRBase version 20 *Homo sapiens*, -v 1 –noRC (27), (iv) Ensembl ncRNAv70 *Homo sapiens*, -v 1 –noRC (28); (v) fRNAdb version 3.4 *Homo sapiens*, -v 1 –noRC (29); (vi) human genome 19, -v 2. miRNA sequences were given a -5p or -3p designation if they aligned with the 5′ or 3′ stem region, respectively, of the miRNA precursor, as annotated in miRBase version 21. miRNAs that represented ≥0.1% of the total miRNA population in both cellular and virion libraries were considered significant (14). Reads for sequences aligning with two or more entries in a database were distributed equally between or among the entries.

### miR-155, miR-92a, and HIV-1 RNA levels in cells and virions determined by qRT-PCR.

HIV-1 was produced by transfecting 10 µg of the HIV-1 proviral plasmids (pNL-NLuc-RAN, pNL-NLuc-155BT, and pNL-NLuc-92aBT) into 293T cells with PEI. In parallel, 2 × 10^6^ 293T cells were transfected with pCMV/CXCR4 (2 µg), pCMV/CD4 (8 µg), and either pcDNA (5 µg) or pmiR-155 (5 µg) to generate 293T cells transiently expressing HIV-1 receptors CD4 and CXCR4 or the receptors plus miR-155. Seventy-two hours posttransfection, virus-containing supernatants were passed through a 0.45-µm filter and used to infect CEM-SS, 293T+CD4/CXCR4, and 293T+CD4/CXCR4/miR-155 cells. Infected cultures were washed with PBS at 24 h postinfection, and at 72 h, the total RNA was isolated from both virions and infected cells with TRIzol. Relative levels of HIV-1, miR-155, and miR-92a were determined as follows. A 500-ng sample of virion or cellular total RNA was subjected to RQ1 RNase-Free DNase treatment (Promega). To determine HIV-1 transcript levels, 250 ng of total RNA was reverse transcribed with SuperScript IV (catalog no. 18090010; Thermo Fisher) according to the manufacturer’s instructions. The samples were diluted 1:5, and 5 µl of each sample was PCR amplified with a custom HIV-1 *pol* TaqMan probe (Applied Biosystems). The remaining 250 ng of DNase-treated RNA was diluted to 5 ng/µl, and 5 µl (25 ng) was reverse transcribed and PCR amplified according to the manufacturer’s protocol with the TaqMan miR-155 (ID002623) and miR-92a (ID000431) miRNA assays. Endogenous U6 RNA levels in cellular RNA samples were determined with the TaqMan RNU6 assay (ID001973). All quantitative PCRs were performed in triplicate in a StepOnePlus real-time PCR system. miR-155 and miR-92a levels relative to HIV-1 strands in virions were calculated by the ΔΔ*C*_*T*_ method (30). miRNA levels in uninfected cells and miRNA and HIV-1 RNA levels in infected cells were calculated relative to the U6 internal control by the ΔΔ*C*_*T*_ method.

### Quantification of miR-155 molecules per HIV-1 strand in virions.

A section of the HIV-1 *pol* gene was PCR amplified with HIV-1-specific primers 5′ AAAGGAAAAAGTCTACCTGGCATGGGTACCAGCAC 3′ (coding) and 5′ ATACATATGGTGTTTTACTAATCTTTTCCATGTGT 3′ (noncoding) and gel purified. Tenfold serial dilutions in CEM-SS cell total RNA (5 ng/µl) of a known concentration of HIV-1 *pol* DNA were PCR amplified by using the custom HIV-1 *pol* TaqMan probe to generate an HIV-1 standard curve. Tenfold serial dilutions in CEM-SS total RNA (5 ng/µl) of a known concentration of an RNA oligonucleotide encoding miR-155 (5′ UUAAUGCUAAUCGUGAUAGGGGU 3′; Integrated DNA Technologies) were amplified by using the TaqMan miR-155 miRNA assay to generate the miR-155 standard curve.

Virus production and virion purification from pNL-NLuc-RAN- and pNL-NLuc-155BT-infected cultures were performed as described above. RNA was isolated from purified virions, and TaqMan qRT-PCR was performed. The experimental *C*_*T*_ values generated were used to calculate absolute values for both miR-155 and HIV-1, which were then used to calculate the number of miR-155 copies per HIV-1 strand in purified virions.

Vesicular stomatitis virus G protein (VSV-G)-pseudotyped pNL-NLuc-RAN and pNL-NLuc-155BT were generated in 2 × 10^6^ 293T cells engineered to stably express miR-155 by PEI transfection with the addition of 2 µg of pVSV-G to each transfection. Pseudotyped HIV-1 virion-containing supernatants were collected at 48 h posttransfection and filtered, and virions were purified by centrifugation as described above. miR-155 and HIV-1 RNA levels were then determined.

### Viral replication assays.

HIV-1 was produced by transfection of 10 µg of the various proviral HIV-1 clones (pNL-NLuc-RAN, pNL-NLuc-155BT, pNL-NLuc-155PT, pNL-NLuc-92aBT, and pNL-NLuc-92aPT) into 293T cells by the PEI method. At 72 h posttransfection, the HIV-1-containing supernatants were used to infect CEM-SS, 293T+CD4/CXCR4, or 293T+CD4/CXCR4/miR-155 cells. Seventy-two hours postinfection, the cells were harvested, washed twice in PBS, and analyzed for NLuc activity with the Nano-Glo luciferase assay kit (Promega).

VSV-G-pseudotyped pNL-NLuc-RAN and pNL-NLuc-155BT were prepared as described above, and p24 levels were determined with an HIV-1 p24 enzyme-linked immunosorbent assay (Advanced Bioscience Laboratories). Naive 293T cells were infected with equivalent amounts of HIV-1 (100 ng p24/ml), and cells were assayed for NLuc production at 20 h postinfection.

### Capsid (p24) and actin Western blot assays.

Virus producer cells were lysed in 1× SDS loading buffer containing 5% 2-mercaptoethanol. Purified virions were diluted in an equal volume of 2× SDS loading buffer containing 5% 2-mercaptoethanol. p24 was detected by Western blotting with an anti-HIV-1 p24 Gag mouse monoclonal antibody (31) (catalog no. 6458; NIH AIDS Reagent Program). Expression of the β-actin loading control was detected with anti-β-actin antibody sc-4778 (Santa Cruz). After being probed with the primary antibodies, the blots were washed and incubated with anti-mouse IgG–horseradish peroxidase (A9044; Sigma-Aldrich). The membranes were washed again and incubated with the WesternBright Sirius Western blotting detection kit (K-12043-D20; Advansta). Chemiluminescence was visualized with the G:BOX Imaging System and GeneSys software (Syngene). p24 and β-actin levels were then quantified with GeneTools (Syngene)

### Statistical analysis.

Statistical significance was assessed with a two-sample, two-tailed, unpaired Student *t* test for populations with unknown, unequal variances. An alpha value of 0.05 was used.

### Accession number(s).

The raw sequencing data obtained by small RNA deep sequencing have been submitted to the NCBI Gene Expression Omnibus and are available under GenBank accession number GSE89868.
