# Supplementary material for: Induced Packaging of Cellular MicroRNAs into HIV-1 Virions Can Inhibit Infectivity
Source: mBio. 2017 Jan 17;8(1):e02125-16. doi: 10.1128/mBio.02125-16 (PMC5241401; doi:10.1128/mBio.02125-16)
Supplement: TABLE S1 [file mbo002173149st1.docx]

| **microRNA** | **Avg. Cell %** | **Avg. Virion %** | **Avg. Fold Enrichment** |
| --- | --- | --- | --- |
| miR-181a-5p | 18.02 | 20.22 | 1.12 |
| miR-191-5p | 16.32 | 13.34 | 0.82 |
| miR-92a-3p | 8.15 | 9.55 | 1.17 |
| miR-16-5p | 4.14 | 3.62 | 0.87 |
| miR-30d-5p | 3.67 | 3.71 | 1.01 |
| miR-142-5p | 2.95 | 2.48 | 0.84 |
| miR-30e-5p | 2.38 | 1.23 | 0.52 |
| miR-378a-3p | 2.01 | 1.83 | 0.91 |
| miR-181b-5p | 1.70 | 2.27 | 1.34 |
| miR-103a-3p | 1.69 | 2.48 | 1.47 |
| miR-93-5p | 1.69 | 1.55 | 0.92 |
| miR-25-3p | 1.55 | 1.23 | 0.79 |
| miR-10a-5p | 1.49 | 1.31 | 0.88 |
| miR-148a-3p | 1.40 | 1.18 | 0.84 |
| miR-182-5p | 1.24 | 1.22 | 0.98 |
| miR-92b-3p | 1.02 | 0.97 | 0.95 |
| miR-30c-5p | 0.99 | 0.69 | 0.70 |
| let-7a-5p | 0.94 | 0.42 | 0.43 |
| miR-301a-3p | 0.91 | 0.62 | 0.68 |
| miR-26a-5p | 0.91 | 0.91 | 1.00 |
