# Supplementary figures and images for: Induced Packaging of Cellular MicroRNAs into HIV-1 Virions Can Inhibit Infectivity
Source: mBio. 2017 Jan 17;8(1):e02125-16. doi: 10.1128/mBio.02125-16 (PMC5241401; doi:10.1128/mBio.02125-16)

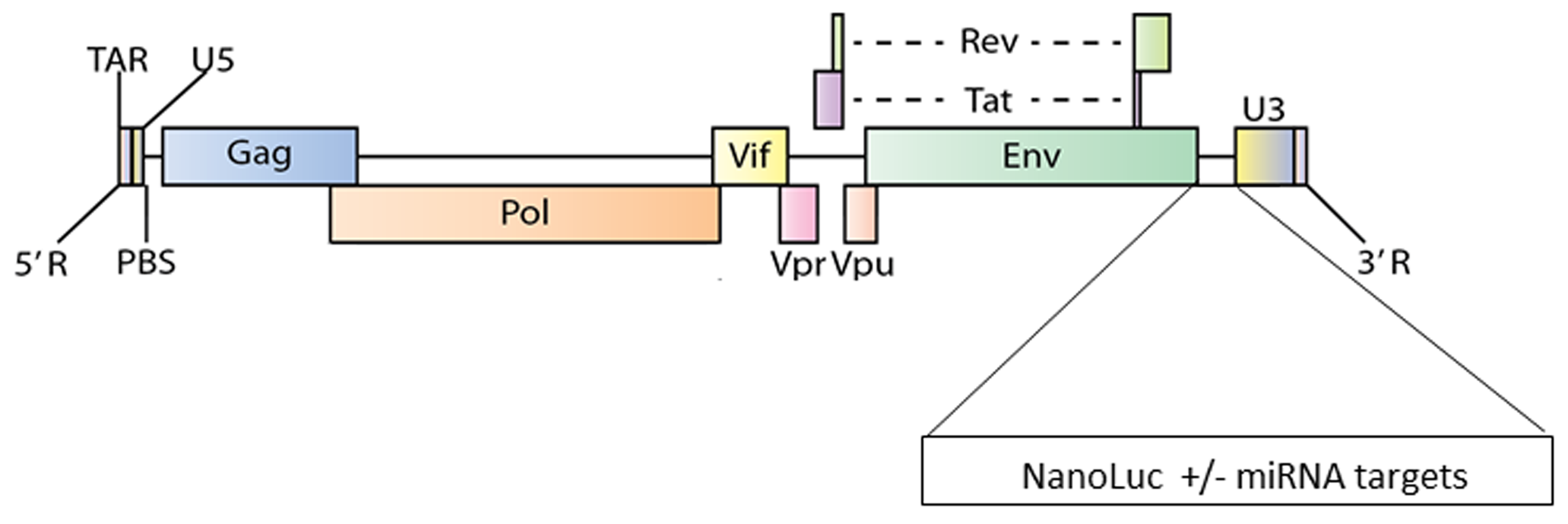

Supplement: FIG S1 [file mbo002173149sf1.tif]

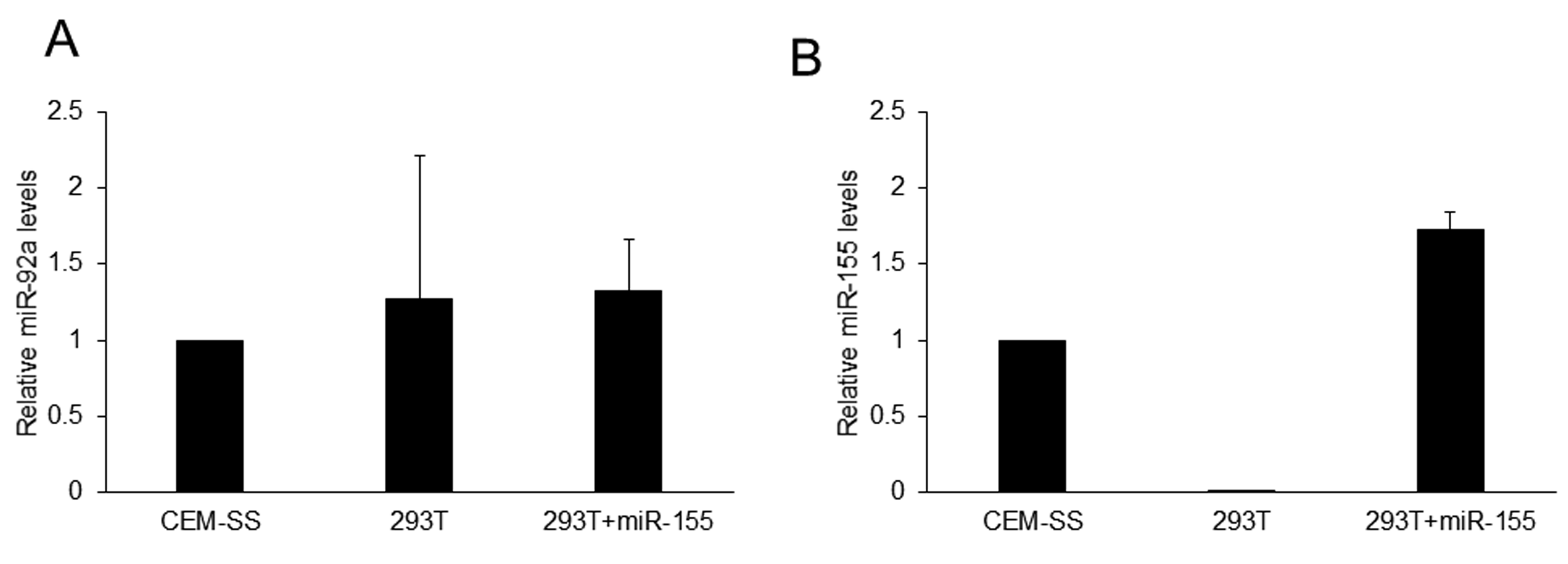

Supplement: FIG S2 [file mbo002173149sf2.tif]

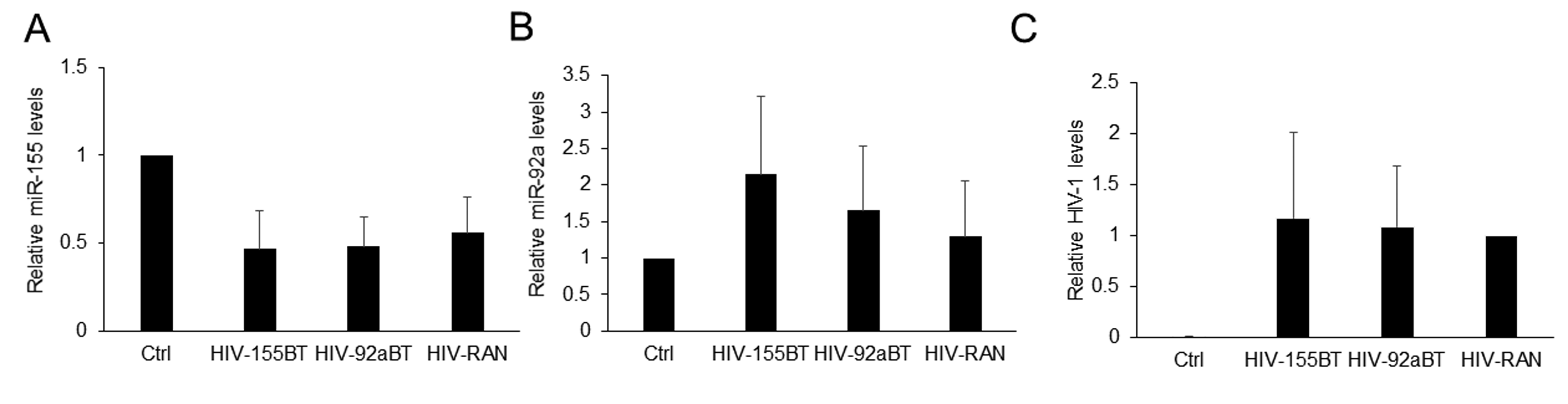

Supplement: FIG S3 [file mbo002173149sf3.tif]
