# Supplementary material for: Induced Packaging of Cellular MicroRNAs into HIV-1 Virions Can Inhibit Infectivity
Source: mBio. 2017 Jan 17;8(1):e02125-16. doi: 10.1128/mBio.02125-16 (PMC5241401; doi:10.1128/mBio.02125-16)
Supplement: FIG S4 [file mbo002173149sf4.pdf]

miR-155:

5′-UUAAUGCUAAUCGUGAUAGGGGU-3′

miR-155PT target:

5′-ACCCCTATCACGATTAGCATTAAggaaaaggACCCCTATCACGATTAGCATTAA-3′

miR-155BT target:

5′-ACCCCTATCA**AT**ATTAGCATTAAggaaaaggACCCCTATCA**AT**ATTAGCATTAA-3′

miR-92a:

5′-UAUUGCACUUGUCCCGGCCUGU-3′

miR-92aPT target:

5′-ACAGGCCGGGACAAGTGCAATAggaaaaggACAGGCCGGGACAAGTGCAATA-3′

miR-92aBT target:

5′-ACAGGCCGGG**TC**AAGTGCAATAggaaaaggACAGGCCGGG**TC**AAGTGCAATA-3′

RAN (random) insert:

5′-CATGCATCTAGAGGGCCCTATTCTATAGTGTCACCTAAATGCTAGAGCTCGCTGGTCG-3′
